# A Risk Profile Calculator for Anterior Cruciate Ligament (ACL) Reconstruction Surgery using a Novel Scoring System: The Multi-factorial ACL Target Score (MATS) Score

**DOI:** 10.5704/MOJ.2507.009

**Published:** 2025-07

**Authors:** M Arora, T Shukla

**Affiliations:** Department of Orthopaedics and Sports Medicine, Fortis Hospital Mohali, Mohali, India

**Keywords:** ACL surgery, ligament surgery, failure, score, tool

## Abstract

**Introduction:**

Anterior cruciate ligament (ACL) surgeries are among the most common orthopaedic surgeries performed globally. The quoted failure rates of ACL surgery are approximately 10-15%, which is unacceptably high. The likely cause of failure is multi-factorial and the ability to predict a high-risk patient pre-operatively will allow surgeons to be better decision makers. The aim of the present study was to assess risk factors for failure and develop a score to help predict failure in ACLR.

**Materials and Methods:**

A retrospective case-control study (n=112 patients) was carried out over a period of two years at a tertiary referral centre. Patients with ACLR failure were grouped into Group 1 (n=56) and patients with a successful ACLR at one year follow-up with no objective or subjective instability AND return to sport were age matched to group 2 (n=56). Risk factor regression analysis was carried out to develop a scoring system (MATS score) and ROC curve analysis was used to generate a cut-off score to predict failure risk.

**Results:**

The frequency mapping data showed a high level of prevalence of risk factors in the test group versus the control group. We found an average MATS score of 4.1 in the control group versus 5.9 in the test group. ROC curve analysis showed that a cut off value of 5.5 may be taken with a good sensitivity and specificity, and good inter-observer reliability.

**Conclusion:**

Based on our assessment of risk factors in the study population we developed the MATS score to aid in clinical decision making. Patients with a score of less than or equal to 5 can be classified as low risk of failure. Patients with a score of 6 or more are considered high risk for ACLR failure.

## Introduction

Anterior cruciate ligament (ACL) reconstruction is one of the most commonly performed procedures worldwide, with an incidence rate between 36.9 and 60.9 per 100000 persons/year^1^. The majority of ACL reconstruction patients have good to excellent outcomes after surgery, however the re-rupture rate of approximately 10-15% of patients^2^ has been a cause for concern.

No universally accepted definition exists for what constitutes failure in ACL surgery. Noyes and Barber-Westin (2001) defined failure as: complete graft tear with >6mm of anterior displacement comparted to the healthy knee; a positive pivot shift test graded +2 or +3 compared to the healthy knee or subjective sensation of instability of functional limitations^3^. Alford and Bach (2005) reported that a more than 3mm difference in antero-posterior knee laxity compared to the healthy knee or an absolute displacement of more than 10mm constitute ACL graft failure with a 99% sensitivity^4^.

So, what causes the high failure rates? In the recent past, a few systematic reviews have tried to answer this question and define risk factors strongly associated with graft failure^5-7^. What is clear is that there is no simple answer, a combination of risk factors are most likely involved that precipitates failure. Having foresight to predict a high failure risk for primary ACL surgery will help surgeons to decide whether adding additional procedures, such as lateral extra-articular procedures (LEAP) or augmenting ACL grafts (biologically or mechanically) may help reduce the failure risk^8^. This should theoretically reduce the failure rate of ACL surgery.

The purpose of the present study was to use risk factor regression analysis for ACL failure in our patient cohort and accordingly develop a scoring tool to aid clinical decision making and calculation of risk for failure.

## Materials and Methods

Ethics approval was obtained from the institutional ethics review committee (IRB: 2021/18-July 2021). A retrospective case-control study was performed over a period of two years (August 2020 – August 2022). All patients entering our service, a tertiary referral hospital, were divided into two groups. Written informed consent was taken from all patients prior to participation in the study. The first group (n=56 patients) were those that had an ACLR performed by either the authors (MA and TS) or referred from another centre and had an ACL failure. ACL failure was defined as subjective instability with objective instability (clinical testing of ACL revealed more than 6mm displacement compared to the contralateral knee) and MRI evidence of partial or complete graft failure^3^. Any patient with objective instability without subjective instability was excluded. Any patient with a side-side difference less than 6mm was excluded, as were patients with multi-ligament knee injuries.

The control group (n=56 patients) were age matched controls who had undergone a successful ACLR during the same interval with a minimum of one year follow-up and had returned to sport. Excluded were patients with any evidence of subjective or objective instability and any patients that had not completed a one-year follow-up or had not returned to sports at some level. Patients with multi-ligament injuries, or those that had undergone an additional procedure such as LEAP or osteotomy were also excluded. An MRI was conducted of all patients to ensure ACL graft was intact.

To ensure pathology matching between both groups, we included only patients with either ACL graft tear with or without meniscal pathology. Any patients with associated osteoarthritis or chondral pathologies requiring intervention were excluded.

Using a correlation coefficient of 0.03 to study the null hypothesis, we found that the sample size should be 80 patients for a moderately high power and reduced error rate. Using 112 patients, the alpha score is <0.03.

One of the authors (TS) was responsible for data collection to eliminate bias. A master chart of risk factors for ACL failure based on the literature was created and all patients were followed-up physically to assess for criteria. Basic demographic data (age, gender, sport) were collated for matching purpose. Risk factors were calculated both for their time of index surgery and for the present duration since the index surgery. Frequency mapping of risk factors and risk factor regression analysis was conducted to aid score creation.

Based on the frequency mapping data and risk factor regression analysis, weightage was given to risk factors according to their presence in Group 1. Accordingly, the scoring system was developed (Table I). The score for each patient in Group 1 and Group 2 were retro calculated to determine MATS score at the time of index surgery.

**Table I:** MATS score with listing of variables for assessment and the weightage given to each variable. The maximum score is out of 22 with a minimum score possible of 0.

	0	1	2	3
Joint hyperlaxity	Beighton <5	Knee hyperextension more than 10 degrees	Beighton >5	
Player/Non-player	Non-player	Recreational	Collegiate	Elite
High contact Sport	No	-	Yes	
Old ACL injury > 1 year (less	Fresh injury than 3 months)	3-12 months old	More than 12 months old	
High grade pivot shift	Negative	Grade1	Grade 2	Grade 3/ Explosive
ACL injury/Reconstruction	Nil	Previous ACL injury without surgery	Previous contralateral ACL reconstruction	Failure of Previous Ipsilateral Reconstruction
Meniscal Deficiency	No ramp	-	Ramp lesion	
Ramp Lesion Increased posterior tibial slope > 12 degrees	No	-	Yes	
Family history of ACL Injury	No	Yes		
BMI	Less than 30	-	More than 30	
Total				

All data related to the study was maintained electronically by TS and entered at each visit. Data set was extracted at the end of the study by another author blinded to the grouping (MA) and divided into variables for analysis. Grouped variables for the MATS score were completed and analysis was carried out using Cochrane’s Q test and McNemar’s test. Cochrane’s Q test is used to determine if there is a difference in dichotomous variables between three or more related groups in a longitudinal study design. A p value <0.05 is used to quote statistical significance. McNemar’s test is used as a post-hoc test dividing the groups into pairs and the final resultant analysis is also divided into pairs to elicit differences. In our series, the number of pairs we compare is 2 or 3 so with a p-value of 0.05 the alpha value becomes 0.05/2 (=0.025) or 0.05/3 (=0.017). Hence with this test, a p-value <0.025 or <0.017, respectively is used for assessment of significance depending on if the variable had 2 or 3 outcomes.

The chi-square or Fisher’s exact test was used for single factor analysis of categorical variables. Single factor analysis of continuous variables was performed with the t-test, receiver operating characteristic curve (ROC curve), and the Youden index (YI); measurements corresponding to the maximum YI value were considered the best diagnostic values. All risk factors were analysed via multivariate logistic regression analysis, and regression coefficients and odds ratios (OR) were calculated for independent risk factors.

ROC curve analysis was used for accuracy prediction of the model method. Patients were scored by the main author (MA) for each patient in group 1 and group 2 and all patients scores were entered into the ROC analysis by second author (TS). Area under the curve was calculated using specificity and sensitivity analysis with tie method. The smallest cut-off was the minimum test value minus 1 and the largest cut-off value was the maximum test value plus 1. All the other cutoff values were the averages of two consecutive ordered observed test values. Kappa value was calculated to test the strength of the relationship.

To assess the inter-observer reliability, we selected 58 patients (29 from Group 1 at random and 29 from Group 2 at random). The score was sent to 2 previous fellows of the institute who were taught how to use the score, and they were instructed that they will receive patient data for 58 patients (blinded to success or failure of ACLR) and they must rate the score for each patient.

## Results

A total of 112 patients were included for analysis, Group 1 (n=56) and Group 2 (n=56), all patients were followed-up till study completion (minimum follow-up duration 1 year). The demographic data is presented in Table II for both groups. Using McNemar’s test and Cochrane’s Q test for statistical significance we found that there is matching for these variables between the test group and control group.

**Table II:** Demographic data comparing the test and control groups using McNemar’s test and Cochrane’s Q test.

	Group 1 test group (n=56)	Group 2 control group (n=56)	p-value
Age (mean years)	25.3	24.8	>0.05
Gender (mean males)	91%	88%	>0.05
Player (mean yes)	92%	94%	>0.05

**Fig. 1:**
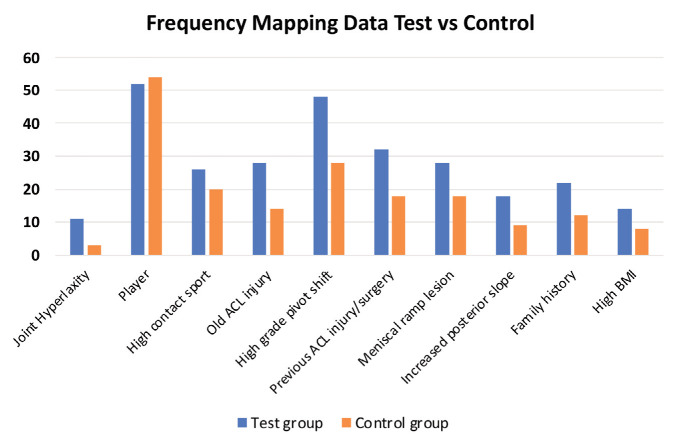
Frequency mapping data for test and control groups for each study variable.

Frequency mapping and risk factor regression analysis was undertaken for both groups with frequency mapping data presented in Fig. 1. There was a higher and statistically significant (p<0.05) frequency for all variables except player/non-player which was more common in the control group and high contact sport which was not significant between the groups.

The frequency mapping data specific to the test group is presented in Table III and risk factor regression analysis of test group is presented in Table IVs. The frequency mapping data and risk factor regression analysis was used to construct the MATS score and give weightage to the variables present in the score. The frequency mapping data indicates that almost each variable has a frequency more than 20% in the study population of the test group with some having a frequency of more than 50%. This is a high onset frequency for any study population and hence the importance of these variables to be part of the scoring system. Some variables are also present in high frequency in the control group such as player/non-player and level of play, and high contact sport.

**Table III:** Frequency mapping data of study variables in the test group.

Variable	Negative variable	Positive variable	Total	Total as percentages of cohort
Joint hyperlaxity	Beighton <5 (n=3)	Beighton <5 (n=8)	11	19%
Player	Recreational (n=20),	Elite (n=20)	52	92%
	Collegiate (n=12)			
High contact sport	No (n=8)	Yes (n=18)	26	46%
Old ACL injury	Less than one year old (n=8)	More than one year old (n=20)	28	50%
High grade pivot shift	No (n=12)	Yes (n=36)	48	86%
Previous ACL injury/surgery	No (n=12)	Yes (n=20)	32	57%
Meniscal ramp lesion	No (n=10)	Yes (n=18)	28	50%
Increased posterior slope	No (n=6)	Yes (n=12)	18	32%
Family history	No (n=9)	Yes (n=13)	22	39%
High BMI	No (n=4)	Yes (n=10)	14	25%

**Table IV:** Regression analysis of risk factors/study variables in the test group.

Variable	Regression co-efficient	Standard error	p-value
Joint hyperlaxity	0.49	0.211	0.044
Player	-0.23	0.185	0.15
High contact sport	-0.44	0.204	0.11
Old ACL injury	0.38	0.099	0.06
High grade pivot shift	0.018	0.003	0.001
Previous ACL injury/surgery	0.023	0.008	0.001
Meniscal ramp lesion	0.048	0.011	0.02
Increased posterior slope	0.203	0.102	0.02
Family history	0.106	0.062	0.04
High BMI	0.281	0.098	0.09

The risk factor regression analysis data shows that the variables most significantly associated with failure are high grade pivot shift and previous ACL surgery or injury. The variables least associated with failure are player/non-player and high contact sport.

**Fig. 2:**
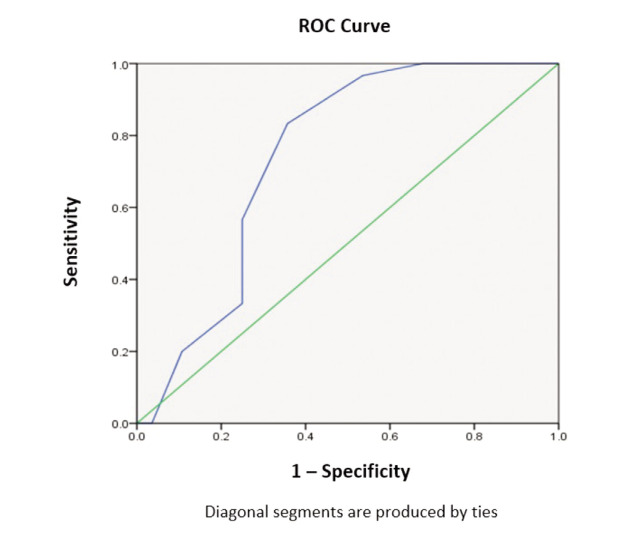
ROC curve analysis of MATS score, green line represents the curve standard and blue line represents MATS score for the test group population (Group 1).

ROC curve was used for accuracy prediction using the model method (Fig. 2 and Table V). The average total MATS score for ACLR success was 4.1 (range: 0-6) while the average score for ACLR failure was 5.9 (range: 3-10). The AUC (area under the curve) analysis value was 0.743. This means the model accurately predicts 74.3% of successful outcomes. Hence, it does a fair job of predicting whether the surgery will be a success or a failure with a p-value of 0.002.

**Table V:** Sensitivity and specificity data for cut off values taken as 0.5 increments from a minimum score of 0 to an observed maximum score of 10.

	Coordinates of the Curve Test Results Variable(s): Total Score		
Positive if Less Than or Equal To	Sensitivity	1 - Specificity	Specificity
0	0	0	1
1.5	0	0.036	0.964
2.5	0.2	0.107	0.893
3.5	0.333	0.25	0.75
4.5	0.567	0.25	0.75
5.5	0.833	0.357	0.643
6.5	0.967	0.536	0.464
7.5	1	0.679	0.321
8.5	1	0.893	0.107
10	1	1	0

Cut-off 5.5 can be considered as the best cut-off which can be used for classifying the outcomes as whether the surgeries will be a success or not as Youden's Index is maximum i.e., 0.476 for this cut-off. For the best cut off 83.3% cases will be correctly classified as successful surgeries i.e., they don’t need any further surgery while 35.7% of the failure surgeries will be incorrectly classified as successful surgeries.

Rater 1 and Rater 2 reliability data for the subset of 58 patients (29 from group 1 and 29 from group 2) is presented in Table VI. Good to strong level of agreement was seen between both raters with a kappa value of 0.828 with statistical significance of <0.005.

**Table VI:** Frequency data for reliability calculation between Rater 1 and Rater

Count		Rater 1 * Rater 2 Cross Tabulation Rater 2		Total
		Failure	Success	
Rater 1	Failure	26	5	31
	Success	2	25	27
Total		28	30	58

## Discussion

ACL reconstruction is one of most commonly performed procedures worldwide, however the failure rate is unacceptably high at 10-15% as per literature estimates^2^. Some recent systematic reviews have tried to define risk factors strongly associated with graft failure^5-7^, which include but are not limited to athletes engaged in high contact sports, those with explosive pivot shift tests, those with increased posterior tibial slope and associated ramp lesions. Based on the risk factors identified in the literature showing a strong correlation with ACLR failure, we performed the present retrospective case-cohort study to undertake frequency mapping and risk factor regression analysis in our study population.

Almost all risk factors studied showed a prevalence of more than 20% in the test group, with some such as being a player and having high grade pivot shift showed almost 90% prevalence. We found that there were certain risk factors which showed a strong and significant correlation with ACLR failure and were prevalent in a high frequency in the ACLR failure group, especially the presence of a high-grade pivot shift and previous ACL injury to the contralateral knee or previous ACL surgery of the contralateral knee. These are factors which have accordingly been given more weightage in the new scoring system as compared to other factors. Some risk factors, such as high BMI had a lower prevalence with a lower correlation and hence were weighted lower in the new scoring system. Accordingly, the scoring system has been developed to provide more weightage to more significant risk factors with higher frequency and less weightage to less significant risk factors with lower frequencies.

One corollary is the athlete. Both the test (92%) and control group (94%) had a high frequency of athletic participation and as such it is hard to determine the true significance of being an athlete on the outcome of ACLR, we know from the literature that the ACLR failure rates are higher in athletes^9^. So, despite not having significance in regression analysis, purely based on frequency metrics we have included the level of athletic play in the scoring system. Elite athletes have been given a higher weightage whereas non-players and recreational athletes have been given a lower weightage.

Based on the above risk factor regression analysis and weightage characteristics, we developed the MATS score (named after the authors of the study). We believe that the MATS score encompasses the majority of the most prevalent risk factors both in our study and the global literature. Some authors have previously attempted to correlate various characteristics such as graft size and diameter with failure risk and develop scoring algorithms to predict failure^10^. The MATS score is different as it does not focus on the surgical aspect rather is patient centric and assesses patient characteristics which are risk factors for failure.

The MATS score analysis data between the study groups with ROC analysis revealed that a cut-off score of 5.5 will allow for a good sensitivity and specificity while capturing a fair area under the curve. This means that if a patient has a score of 5 or less, we can consider them as a low risk of failure. If a patient has a score of 6 or more, they may be considered as a high failure risk and here the decision can be taken to add a LEAP procedure or augment the ACL graft to reduce the failure risk^8^ in addition to performing the individual procedures such as ramp repair for ramp lesions etc.

Inter-observer reliability data shows that with a high kappa value there is a good degree of agreement between scorers and hence the MATS score is reliable to use. This is due to the presence of primarily objective end points in the scoring system and no subjective points which would lead to mismatch or disagreement. On a larger scale usage, we believe that the objective end points of the scoring system will allow for a more widespread and reliable application.

The MATS score is by no means an end all or encompassing all scores for ACL failure risk. The purpose of the score is to provide an easy to use and reproducible scoring system to aid decision making. Hence it suffers from many limitations. Firstly, the list of risk factors included is not comprehensive. Many other factors drive ACL graft failure, such as notch stenosis and graft diameter, have not been included to keep the score concise and prevent an exhaustive list which becomes cumbersome for the surgeon. A major area which was been excluded is the multiligament injury. We know that PLC injuries are associated with higher ACL failure rates^11^, as are neglected MCL injuries^12^, however the assessment of each has been excluded from the score again due to conciseness. We advocate that in the setting of a multi-ligament knee, the MATS score to not be applied and each ligament to be treated on its own merit.

The true assessment of a score lies in the fact that does it actually do what it is intended for. Does the MATS score actually reduce the graft failure rate? We are conducting a longitudinal cohort study of patients since the implementation of the MATS score which aims to answer this very question. Another fallacy of the development of the scoring system related to the presence or absence of a ramp lesion. The study was limited in the fact that for patients coming for revision surgery done by another surgeon we were reliant on the pre-operative MRI images or the surgical notes to determine presence or absence of a ramp. This may have skewed the ramp data. At this stage, we recommend the MATS score as only a tool to aid surgical decision making and not be the sole criteria to decide failure.

## Conclusion

ACL surgeries fail at an alarmingly high rate which is likely multi-factorial. We performed a retrospective case-control study to identify patient risk factors and their frequency based on the prevailing literature. Accordingly, we have developed the MATS score, a tool to aid surgical decision making, classifying patients as high or low risk for ACLR failure.
